# Trajectories of Kidney Function in Autosomal Dominant Polycystic Kidney Disease Patients Treated with Tolvaptan

**DOI:** 10.3390/medicina62010194

**Published:** 2026-01-16

**Authors:** Zofia Jankowska, Mariusz Niemczyk

**Affiliations:** Department of Transplantology, Immunology, Nephrology, and Internal Diseases, Medical University of Warsaw, 02-006 Warszawa, Poland

**Keywords:** autosomal dominant polycystic kidney disease, trajectories of eGFR, tolvaptan

## Abstract

*Background and Objectives:* Autosomal dominant polycystic kidney disease (ADPKD) is the most common monogenic kidney disease, and the only approved pharmacological therapy shown to slow disease progression is tolvaptan. This study presents a long-term observation of ADPKD patients treated at our center, focusing on changes in eGFR approximately one year before and at least 1 year after the initiation of tolvaptan therapy. *Materials and Methods:* A retrospective analysis of a cohort of ADPKD patients who have received tolvaptan treatment in our center. *Results:* In total, 20 patients were enrolled in the analysis. Their median time of observation since tolvaptan introduction was 23.5 months. No statistically significant difference was noted in the median monthly decrease in eGFR between the time prior to tolvaptan introduction and during tolvaptan therapy. Analysis of trajectories of eGFR in particular patients enabled the division of the cohort into three subgroups: beneficiaries (n = 7, 35%), stable (n = 8, 40%), and progressors (n = 5, n = 25%). *Conclusions:* Despite the low number of patients, together with a relatively short observation period, which are the main limitations of our study, our results suggest that, in real-world settings, the efficacy of tolvaptan may be lower than previously reported. There is an urgent need to identify factors responsible for the suboptimal effect of the medicine. Our findings underscore the need to re-evaluate the current inclusion criteria for tolvaptan, particularly in real-world settings where patient variability is broader than in controlled clinical trials. Tailoring treatment qualification to include more practical and region-specific factors may enhance therapeutic outcomes.

## 1. Introduction

Autosomal dominant polycystic kidney disease (ADPKD) is the most common monogenic kidney disease, with a prevalence of approximately 1 in 1000 individuals [1]. It often leads to end-stage kidney disease in adulthood [2]. The condition is most often caused by mutations in the *PKD1* gene, which affects polycystin-1, or, less commonly, in *PKD2*, which encodes polycystin-2. In rare cases, mutations in other genes may be involved in disease pathogenesis, e.g., *IFT140*, *DNAJB11*, or *GANAB* [3].

The pathophysiology of ADPKD involves the gradual development of multiple cysts in both kidneys and the progression of chronic kidney disease (CKD). The decline of kidney function is best monitored using the estimated glomerular filtration rate (eGFR), the most reliable marker of kidney function [3].

Currently, the only approved pharmacological therapy shown to slow disease progression is tolvaptan, a selective vasopressin V2 receptor antagonist [4]. Its efficacy has been demonstrated in clinical control studies such as TEMPO 3:4 [5] and REPRISE [6]. However, studies like the one by Borowiecka et al. [7] have raised questions about the applicability of large clinical trial inclusion criteria to real-life patient populations. This is especially relevant in light of the potential adverse effects of tolvaptan, such as hepatotoxicity, and the practical inconvenience associated with its use [8].

This study presents a long-term observation of ADPKD patients treated at our center, focusing on changes in eGFR for at least one year before and after the initiation of tolvaptan therapy.

## 2. Materials and Methods

Our study was a retrospective analysis of a cohort of ADPKD patients who have received tolvaptan treatment in one of the outpatient clinics of the Department of Transplantology, Immunology, Nephrology, and Internal Diseases of the Medical University of Warsaw, Poland. The treatment was initiated between September 2022 and October 2024.

The patients received tolvaptan according to the Polish refund criteria:

- Diagnosis of ADPKD is established through imaging techniques (ultrasound, computed tomography, or magnetic resonance) and/or genetic testing.
- Age: Eighteen years and above.
- Evidence of rapid disease progression:
  ○ An annual decline in eGFR of ≥5 mL/min/1.73 m^2^ in patients with eGFR between 30 and 90 mL/min/1.73 m^2^;
  ○ An annual decline in eGFR of ≥2.5 mL/min/1.73 m^2^ during the last 5 years in patients with eGFR between 30 and 60 mL/min/1.73 m^2^;
  ○ An annual increase in total kidney volume (TKV) assessed with magnetic resonance (MR) of > 5% or TKV of one kidney assessed with MR of >750 mL, or length of a larger kidney in ultrasound of >16.5 cm.
- Absence of contraindications:
  ○ Liver disease or elevated liver enzymes;
  ○ Inability to adhere to the required monitoring protocols;
  ○ Pregnancy or breastfeeding;
  ○ Hypovolemia or hypernatremia.

All patients were treated by the same nephrologist and were informed about the dosage and necessary fluid intake during the therapy. The patients’ long-term pharmacotherapy other than tolvaptan was continued as usual. The dose of tolvaptan was 60 mg daily in the first 4 weeks, 90 mg daily in the subsequent 4 weeks, and 120 mg daily starting from the beginning of the 9th week of therapy. If required, the target dose was decreased.

Patients with at least a 1-year-long follow-up were included. Patients who discontinued tolvaptan within 1 year of observation due to the side effects or discontinued per the patients’ request were excluded from our analysis.

We recorded the following data for each patient:

- Age at the time of the initiation of tolvaptan therapy, sex, race, height, weight, blood pressure, and kidney length;
- Self-reported daily fluid intake during therapy;
- Medicines used;
- Laboratory results of blood test: Serum creatinine from each consecutive visit (within approximately 1 year prior to administration and on tolvaptan), sodium, potassium, hemoglobin, C-reactive protein (CRP), and glucose;
- In urine: Urine-specific gravity and presence of proteinuria in the urinalysis.

The eGFR was calculated using the CKD-EPI 2021 formula. Rate of CKD progression prior to tolvaptan introduction was assessed as the difference between the average eGFR in approximately 1 year prior to therapy and eGFR at the moment of therapy initiation. The rate of CKD progression on tolvaptan was assessed as the difference between eGFR at the moment of initiation of therapy and the last available eGFR on tolvaptan. Graphs illustrating trajectories of eGFR in the pre-treatment period, as well as on treatment, were performed to enable visual assessment of eGFR steepness.

Statistical analysis was performed using Statistica 13.3 (StatSoft, Tulsa, OK, USA). Normality of data distribution was assessed with the Shapiro–Wilk test. The chi-squared test was used for comparison of categorical data. For comparison of continuous data, the U Mann–Whitney test or the Wilcoxon signed rank test was used when appropriate. Categorical data were expressed as absolute data with percentages, while continuous data were presented as medians, ranges, and interquartile ranges (IQRs). Due to the relatively low number of cases, results with *p* < 0.1 were considered statistically significant.

The study was conducted according to the principles of the Declaration of Helsinki. The local Ethics Committee was informed about the study (confirmation no. AKBE/51/2024). Due to the character of the study, patients’ written informed consent was redundant.

## 3. Results

Between September 2022 and October 2024, treatment with tolvaptan was started in 26 patients. In six patients, the medicine had to be discontinued within the first year of therapy due to intolerable adverse effects or the patients’ demand.

As a result, 20 patients fulfilled the inclusion criteria and were enrolled in the analysis. Their characteristics are presented in Table 1. All patients were White. The median time of observation since tolvaptan introduction was 23.5 months (IQR 18.5, range: 11–35). At the time of our analysis, 12 patients (60%) were still on tolvaptan, while in 8 patients (40%), tolvaptan was discontinued due to the fact that eGFR decreased below 15 mL/min/1.73 m^2^. In no patient was ACE-I or ARB started or discontinued during tolvaptan therapy. In some cases, however, the daily dose was decreased according to the reduction in renal function.

In the whole cohort, the median change in eGFR was −0.429 mL/min/1.73 m^2^ per 1 month (IQR 0.476, range: from −1.444 to 0.750) prior to tolvaptan introduction, and −0.373 mL/min/1.73 m^2^ per 1 month (IQR 0.303, range: from −1.364 to 0.333) during tolvaptan therapy; no statistically significant difference was noted in the median monthly decrease in eGFR between time prior to tolvaptan introduction and during tolvaptan therapy (*p* = 0.737, Wilcoxon signed rank test).

Subsequently, analysis of trajectories of eGFR in particular patients enabled the division of the cohort into three subgroups:

- Beneficiaries (n = 7), in whom an advantageous change in the rate of eGFR decline was observed with tolvaptan therapy;
- Stable (n = 8), in which tolvaptan did not lead to a change in the rate of progression of CKD;
- Progressors (n = 5), in which the rate of CKD progression accelerated on tolvaptan.

**Figure 1:**
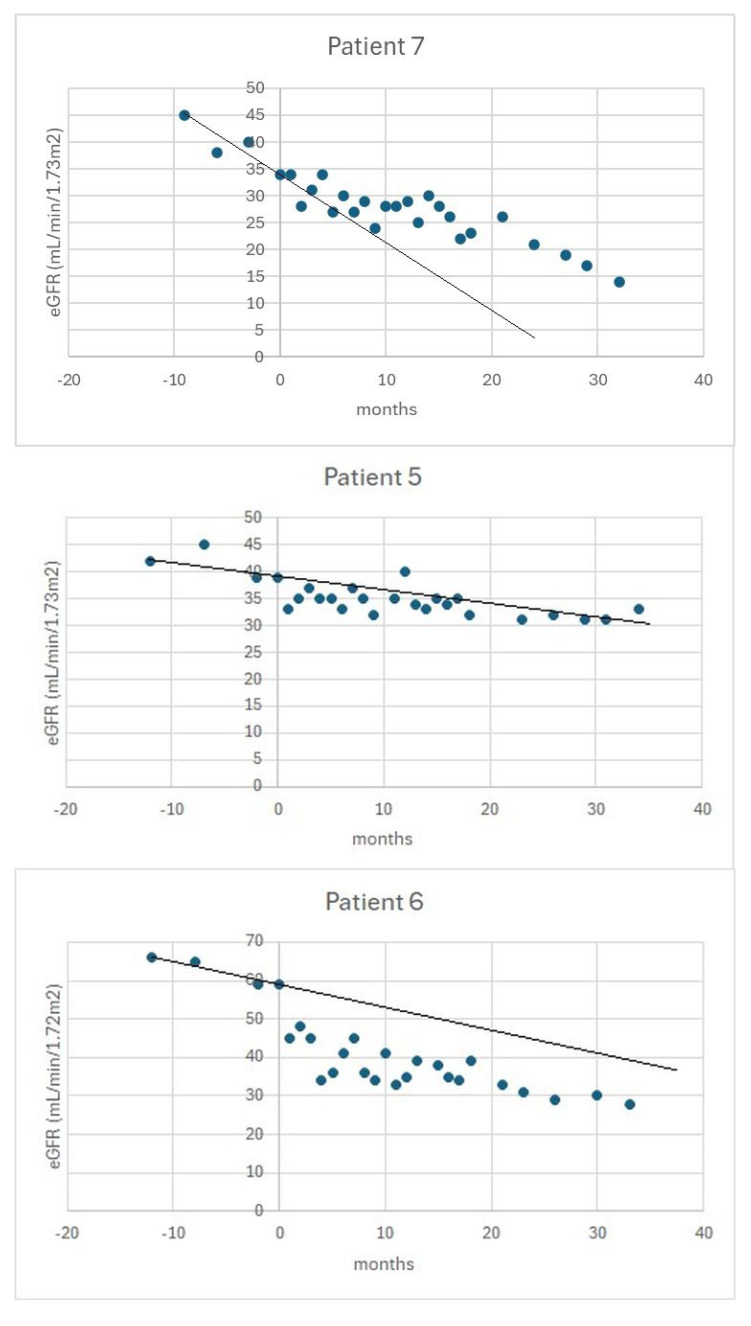
An example of the trajectory of eGFR in a beneficiary (**upper** panel), a stable patient (**middle** panel), and a progressor (**bottom** panel). The vertical axis indicates the time of initiation of tolvaptan. The black line indicates the slope of eGFR in the pre-treatment period.

Figure 1 shows examples of eGFR trajectories in patients from the above subgroups.

Finally, we proceeded to compare characteristics between beneficiaries (n = 7) and non-beneficiaries, id est, combined stable and progressors (n = 13). Table 2 presents a comparison of characteristics between beneficiaries and non-beneficiaries.

## 4. Discussion

Tolvaptan decreases the rate of CKD progression in ADPKD; it has been shown both in clinical trials and in real-world settings [8,9]. Additionally, some benefits have been observed in ADPKD patients treated with tolvaptan: reduction in the nephrolithiasis risk [10], increase in bone mineral density [11], reduction in pain and hematuria events [12], as well as the reduction in inflammation [13] and oxidative stress [14]. Therefore, our results were quite surprising for us; according to our observation, in nearly half of our ADPKD cohort, tolvaptan did not change the rate of CKD progression, while the beneficial effects of tolvaptan on the rate of CKD progression were evident in only 35% of patients treated with the medicine. Moreover, in 25% of the cohort, acceleration of the rate of CKD progression was observed on tolvaptan.

At least in part, our results may be explained by the methods of selection of our cohort. Tolvaptan is recommended for ADPKD patients with fast progression of CKD, and TKV measurements are considered an optimal method of selection of fast progressors [15]. Maximum kidney length assessed in abdominal ultrasound may be used as a marker of fast progression only in cases when TKV measurements are unavailable [4]. However, in the majority of our cohort, maximum kidney length was used instead of TKV in patient selection. It was in accordance with the criteria of the Polish drug prescription program [16], but, in light of our results, it should probably not be widely used. This raises a question of whether more clinically useful criteria should be developed for treatment qualification. On the other hand, the rate of progression of CKD in our cohort prior to the initiation of tolvaptan, with a median accounting to 0.429 mL/min/1.73 m^2^ per 1 month, that is, 5.15 mL/min/1.73 m^2^ per year, met the criteria of fast progression in the majority of the cohort [3,17].

Another explanation of our results could be dehydration, caused by tolvaptan-associated diuresis. High water intake was proven to be also efficient in slowing the progression of CKD in ADPKD patients [18]; however, not as efficient as tolvaptan [19]. Clinical signs of dehydration, as well as natremia, were assessed in our patients at each visit, and dehydration was not observed. However, self-reported daily fluid intake was higher in beneficiaries compared to non-beneficiaries. Therefore, we feel that dehydration may be considered a potential reason of low efficacy of tolvaptan in some patients from our cohort. However, it should be noted that urine osmolality was not assessed in our cohort. Urine osmolality is considered a measure of effect in ADPKD patients treated with tolvaptan [15]. It has been shown that the decrease in urine osmolality predicts the efficacy of tolvaptan [20]. Moreover, urine osmolality is considered a basis for dose titration [21]. Again, based on the rules of the Polish drug prescription program [16], as well as the recommendations of the Working Group of the Polish Society of Nephrology [17], measurements of urine osmolality were waived in our cohort, and doses were not titrated accordingly. As a result, the majority of our patients received the maximum dose of tolvaptan (i.e., 120 mg daily), which could be responsible for unfavorable clinical results. In fact, it was shown that in real-life settings, many patients required lower doses of tolvaptan compared to clinical trials [22].

During our analysis, we attempted to search for factors that might differentiate between those who benefited from tolvaptan therapy and those who did not. We did not show statistically significant differences in the analyzed parameters except for the systolic blood pressure at the moment of initiation of therapy, and self-reported daily fluid intake during therapy, which was discussed above (Table 2). The role of blood pressure was not surprising, as it is well-known that hypertension is closely linked to renal function decline [23]. As expected, age was not differentiated between the groups. It is in line with the literature data, because tolvaptan efficacy was shown to be independent of the patients’ age [24]. Similarly, body mass index had no impact on the effect of tolvaptan, which is also in accordance with the current views [25]. Beneficiaries and non-beneficiaries did not differ in renal function at the moment of initiation of therapy, which was also not surprising, while the efficacy of tolvaptan was proven in patients on different CKD stages [6,26,27]. However, based on the results of Mochizuki et al. [28], we expected that the decrease in eGFR within the first 3 months of therapy could predict the long-term effects of the medicine. However, as shown in Table 2, despite the fact that the median decrease in eGFR during that time was higher in the beneficiaries than in non-beneficiaries, the difference did not reach statistical significance.

Proteinuria is associated with increased risk for the progression of CKD in ADPKD [29]. Therefore, it should be considered an indication for tolvaptan treatment. It was shown that tolvaptan is effective in ADPKD patients with albuminuria and that treatment leads to a decrease in albuminuria [30]. In our cohort, the presence of proteinuria at the initiation of therapy did not impact the outcome. Possibly, to improve the efficacy of tolvaptan in patients with ADPKD and proteinuria, additional options should be considered. Sodium-Glucose Cotransporter 2 (SGLT2) inhibitors are effective in CKD with proteinuria [31], and some reports support improvement of tolvaptan effects when combined with an SGLT2 inhibitor in ADPKD patients with proteinuria [32,33].

Limitations of our study should be acknowledged. First, it was a single-center, retrospective analysis. Second, the number of patients in the cohort was low, yet it was sufficient to demonstrate the effects of tolvaptan in a real-life clinical situation, which was our main objective. Third, the observation period may be considered relatively short. Fourth, only a limited number of factors were analyzed. For example, due to the lack of data, types of mutations in the patients were omitted in the analysis. In fact, the type of the mutation responsible for the disease influences the efficacy of tolvaptan [34]. Moreover, our analysis was based on the assumption that the slope of eGFR is nearly linear, which may not necessarily be true in each case. Finally, the measurement of kidney function based on serum creatinine and eGFR can be potentially inaccurate.

## 5. Conclusions

In real-world settings, the efficacy of tolvaptan may be lower than previously reported. There is an urgent need to identify factors responsible for the suboptimal effect of the medicine. Our findings underscore the need to re-evaluate the current inclusion criteria for tolvaptan, particularly in real-world settings where patient variability is broader than in controlled clinical trials. Tailoring treatment qualification to include more practical and region-specific factors may enhance therapeutic outcomes. Future prospective, multi-center studies are warranted to validate more clinically relevant selection strategies.

## Figures and Tables

**Table 1:** Characteristics of study cohort.

Feature	Result
At initiation of therapy	
Men/women, n (%)	11 (55)/9 (45)
Age at initiation of tolvaptan in years, mean (SD)	43.35 (7.34)
Body mass index in kg/m^2^, mean (SD)	26.99 (3.59)
Systolic blood pressure in mmHg, median (IQR)	130 (9.5)
Diastolic blood pressure in mmHg, median (IQR)	85 (8.0)
Maximum kidney length in cm, median (IQR)	21.75 (3.75)
eGFR at initiation of tolvaptan in mL/min/1.73 m^2^, median (IQR)	35.83 (25.12)
Natremia in mmol/L, mean (SD)	139.9 (2.3)
Kalemia in mmol/L, mean (SD)	4.45 (0.51)
Hemoglobin level in g/dL, mean (SD)	13.58 (1.57)
Serum glucose in mg/dL, median (IQR)	92.5 (7.0)
C-reactive protein in mg/L, median (IQR)	1.05 (1.10)
Urine specific gravity in g/mL, median (IQR)	1.009 (0.005)
Proteinuria, n (%)	6 (30)
During therapy	
Change in eGFR during first 3 months of therapy in mL/min/1.73 m^2^, mean (SD)	−0.178 (6.802)
Self-reported daily fluid intake in liters, mean (SD)	5.93 (1.36)
Medicine use, n (%):	
Statin	12 (60)
ACE-I/ARB	13 (65)
Calcium channel blocker	12 (60)
Beta-blocker	13 (65)
Alpha-blocker	6 (30)
Diuretic	14 (70)
Allopurinol	7 (35)
SGLT2 inhibitors	0 (0)
Aldosterone receptor antagonist	0 (0)
GLP-1 analogs	0 (0)
Mean daily dose of tolvaptan between the end of the 3rd month of therapy and the end of observation in mg, median (IQR)	120 (0.5)

SD: standard deviation; eGFR: estimated glomerular filtration rate; IQR: interquartile range.

**Table 2:** Comparison between beneficiaries and non-beneficiaries.

Feature	Beneficiaries (n = 7)	Non-Beneficiaries (n = 13)	*p*
Men/women, n (%)	4 (57)/3 (43)	7 (54)/6 (46)	*p* = 0.630 *
Age at initiation of tolvaptan in years, median, IQR, range	42, 11, 32–55	42, 6, 35–60	*p* = 0.692 **
Body mass index in kg/m^2^, median, IQR, range	27.50, 6.77, 21.61–32.89	28.06, 3.74, 20.20–33.00	*p* = 0.579 **
Systolic blood pressure in mmHg, median, IQR, range	125, 8, 110–130	130, 10, 110–160	*p* = 0.068 **
Diastolic blood pressure in mmHg, median, IQR, range	85, 5, 60–92	85, 5, 75–110	*p* = 0.250 **
Maximum kidney length in cm, median, IQR, range	22, 3, 19–24	21, 4.5, 17.7–32	*p* = 0.968 **
eGFR at initiation of tolvaptan in mL/min/1.73 m^2^, median, IQR, range	36.49, 24.17, 24.32–66.59	35.17, 27.96, 20.74–93.00	*p* = 0.782 **
boldNatremia in mmol/L, median, IQR, range	140, 5.6, 136.3–144.0	139.9, 2.2, 136.0–143.0	*p* = 0.691 **
Kalemia in mmol/L, median, IQR, range	4.60, 0.70, 3.44–4.87	4.40, 0.54, 3.80–5.51	*p* = 0.905 **
Hemoglobin level in g/dL, median, IQR, range	13.6, 3.0, 10.9–18.0	13.2, 1.3, 11.7–15.2	*p* = 0.721 **
Serum glucose in mg/dL, median, IQR, range	94.7, 7, 82–95	92.8, 8, 81–122	*p* = 0.634 **
C-reactive protein in mg/L, median, IQR, range	1.0, 1.3, 0.8–15.4	1.5, 0.9, 0.6–4.0	*p* = 0.968 **
Urine specific gravity in g/mL, median, IQR, range	1.007, 0.006, 1.003–1.011	1.009, 0.005, 1.005–1.022	*p* = 0.267 **
Proteinuria, n (%)	1 (14)	6 (46)	*p* = 0.177 *
Self-reported daily fluid intake in liters, median, IQR, range	6, 1.5, 5–10	5.5, 1.5, 4–7	*p* = 0.057 **
Change in eGFR during first 3 months of therapy in mL/min/1.73 m^2^, median, IQR, range	−2.36, 5.01, −16.84–2.33	−0.75, 10.04, −9.64–12.000	*p* = 0.234 **
Mean daily dose of tolvaptan between the end of the 3rd month of therapy and the end of observation in mg, median, IQR, range	120, 0, 120–120	120, 0, 75–120	*p* = 0.428 **
Time of observation in months, median, IQR, range	24, 20, 12–35	23, 17, 11–34	*p* = 0.905 **
ACE-I/ARB use, n (%)	4 (57)	9 (69)	*p* = 0.474 *

IQR, interquartile range; *, Fisher’s exact test; **, Mann–Whitney U test. Bold denotes statistical significance.

## Data Availability

Detailed study data are available from the corresponding author on demand.

## Abbreviations

The following abbreviations are used in this manuscript:

ACE-I	Angiotensin-converting enzyme inhibitor
ADPKD	Autosomal dominant polycystic kidney disease
ARB	Angiotensin receptor blocker
CKD	Chronic kidney disease
CRP	C-reactive protein
eGFR	Estimated glomerular filtration rate
IQR	Interquartile range
SD	Standard deviation
SGLT2	Sodium-glucose transporter 2
TKV	Total kidney volume

## References

[1] Rangan G.K., I Alexander S., Campbell K.L., Dexter M.A., Lee V.W., Lopez-Vargas P., Mai J., Mallett A., Patel C., Patel M. (2016). KHA-CARI guideline recommendations for the diagnosis and management of autosomal dominant polycystic kidney disease. Nephrology.

[2] Hateboer N., v Dijk M.A., Bogdanova N., Coto E., Saggar-Malik A.K., Millan J.L.S., Torra R., Breuning M., Ravine D. (1999). Comparison of phenotypes of polycystic kidney disease types 1 and 2. European PKD1-PKD2 Study Group. Lancet.

[3] Torres V.E., Ahn C., Barten T.R.M., Brosnahan G., Cadnapaphornchai M.A., Chapman A.B., Gall E.C.-L., Drenth J.P., Gansevoort R.T., Harris P.C. (2025). KDIGO 2025 clinical practice guideline for the evaluation, management, and treatment of autosomal dominant polycystic kidney disease (ADPKD): Executive summary. Kidney Int..

[4] Muller R.U., Messchendorp A.L., Birn H., Capasso G., Gall E.C.-L., Devuyst O., van Eerde A., Guirchoun P., Harris T., Hoorn E.J. (2022). An update on the use of tolvaptan for autosomal dominant polycystic kidney disease: Consensus statement on behalf of the ERA Working Group on Inherited Kidney Disorders, the European Rare Kidney Disease Reference Network and Polycystic Kidney Disease International. Nephrol. Dial. Transpl..

[5] Blair H.A. (2019). Tolvaptan: A review in autosomal dominant polycystic kidney disease. Drugs.

[6] Torres V.E., Chapman A.B., Devuyst O., Gansevoort R.T., Perrone R.D., Koch G., Ouyang J., McQuade R.D., Blais J.D., Czerwiec F.S. (2017). Tolvaptan in Later-Stage Autosomal Dominant Polycystic Kidney Disease. N. Engl. J. Med..

[7] Borowiecka J., Pączek L., Niemczyk M. (2024). Tolvaptan in autosomal dominant polycystic kidney disease—A real-life experience. Med. Res. J..

[8] Raina R., Houry A., Rath P., Mangat G., Pandher D., Islam M., Khattab A.G., Kalout J.K., Bagga S. (2022). Clinical Utility and Tolerability of Tolvaptan in the Treatment of Autosomal Dominant Polycystic Kidney Disease (ADPKD). Drug Healthc. Patient Saf..

[9] Geertsema P., Bais T., Kuiken V., E Knol M.G., Casteleijn N.F., Vart P., Meijer E., Gansevoort R.T. (2025). The long-term effect of tolvaptan treatment on kidney function and volume in patients with ADPKD. Nephrol. Dial. Transpl..

[10] Bargagli M., Dhayat N.A., Anderegg M., Semmo M., Huynh-Do U., Vogt B., Ferraro P.M., Fuster D.G. (2020). Urinary Lithogenic Risk Profile in ADPKD Patients Treated with Tolvaptan. Clin. J. Am. Soc. Nephrol..

[11] Bargagli M., Vetsch A., Anderegg M., A Dhayat N., Huynh-Do U., Faller N., Vogt B., Ferraro P.M., Fuster D.G. (2023). Tolvaptan treatment is associated with altered mineral metabolism parameters and increased bone mineral density in ADPKD patients. Nephrol. Dial. Transpl..

[12] Li X., Li W., Li Y., Dong C., Zhu P. (2023). The safety and efficacy of tolvaptan in the treatment of patients with autosomal dominant polycystic kidney disease: A systematic review and meta-analysis. Nefrol. Engl. Ed..

[13] Sahin A.Z., Ozdemir O. (2025). The effect of tolvaptan on renal progression and systemic inflammation in ADPKD. Sci. Rep..

[14] Rigato M., Carraro G., Cirella I., Dian S., Di Vico V., Stefanelli L.F., Ravarotto V., Bertoldi G., Nalesso F., Calò L.A. (2022). Effects Effects of Tolvaptan on Oxidative Stress in ADPKD: A Molecular Biological Approach. J. Clin. Med..

[15] Chebib F.T., Perrone R.D., Chapman A.B., Dahl N.K., Harris P.C., Mrug M., Mustafa R.A., Rastogi A., Watnick T., Yu A.S.L. (2018). A Practical Guide for Treatment of Rapidly Progressive ADPKD with Tolvaptan. J. Am. Soc. Nephrol..

[16] https://www.gov.pl/web/zdrowie/obwieszczenie-ministra-zdrowia-z-dnia-21-pazdziernika-2021-r-w-sprawie-wykazu-refundowanych-lekow-srodkow-spozywczych-specjalnego-przeznaczenia-zywieniowego-oraz-wyrobow-medycznych-na-1-listopada-2021-r.

[17] Jankowska M., Dębska-Ślizień A., Krajewska M., Krzanowska K., Kurnatowska I., Durlik M., Niemczyk M., Małyszko J., Stompór T., Stróżecki P. (2023). Principles of using tolvaptan in the treatment of patients with autosomal dominant polycystic kidney disease (ADPKD). Recommendations of the Working Group of the Polish Socirty of Nephrology. Ren. Dis. Transplant. Forum..

[18] Gobburu J., Ivaturi V., Wang X., Shoaf S.E., Jadhav P., Perrone R.D. (2023). Comparing Effects of Tolvaptan and Instruction to Increase Water Consumption in ADPKD: Post Hoc Analysis of TEMPO 3:4. Kidney360.

[19] Bankir L., Guerrot D., Bichet D.G. (2023). Vaptans or voluntary increased hydration to protect the kidney: How do they compare?. Nephrol. Dial. Transplant..

[20] Akihisa T., Kataoka H., Makabe S., Manabe S., Yoshida R., Ushio Y., Sato M., Yajima A., Hanafusa N., Tsuchiya K. (2024). Immediate drop of urine osmolality upon tolvaptan initiation predicts impact on renal prognosis in patients with ADPKD. Nephrol. Dial. Transplant..

[21] Akihisa T., Manabe S., Kataoka H., Makabe S., Yoshida R., Ushio Y., Watanabe K., Sato M., Tsuchiya K., Mochizuki T. (2021). Dose-Dependent Effect of Tolvaptan on Renal Prognosis in Patients with Autosomal Dominant Polycystic Kidney Disease. Kidney360.

[22] Rao V., Ammar S., Alshorman A., Fravel M., McGreal K.A., Winklhofer F.T., Noureddine L., Jalal D.I., Yu A.S.L., Mustafa R.A. (2025). Real-World Tolvaptan Use in Autosomal Dominant Polycystic Kidney Disease: Insights from Two US Medical Centers. Kidney360.

[23] Ebrahimi N., Caliskan Y., Garimella P.S., Carriazo S., Chebib F.T., Bateni G.H., Dahl N.K., Rastogi A., Abdipour A., Norouzi S. (2025). Cardiovascular Complications in ADPKD. Kidney Int. Rep..

[24] Chebib F.T., Zhou X., Garbinsky D., Davenport E., Nunna S., Oberdhan D., Fernandes A. (2023). Tolvaptan and Kidney Function Decline in Older Individuals With Autosomal Dominant Polycystic Kidney Disease: A Pooled Analysis of Randomized Clinical Trials and Observational Studies. Kidney Med..

[25] Nowak K.L., Steele C., Gitomer B., Wang W., Ouyang J., Chonchol M.B. (2021). Overweight and Obesity and Progression of ADPKD. Clin. J. Am. Soc. Nephrol..

[26] Torres V.E., Gansevoort R.T., Perrone R.D., Chapman A.B., Ouyang J., Lee J., Japes H., Nourbakhsh A., Wang T. (2021). Tolvaptan in ADPKD Patients With Very Low Kidney Function. Kidney Int. Rep..

[27] Sekine A., Hoshino J., Mochizuki T., Nakatani S., Nishio S., Suwabe T., Hayashi H., Kai H., Seta K., Hattanda F. (2025). Kidney Function Trajectories with Tolvaptan in ADPKD Patients with CKD-G5. Kidney Int. Rep..

[28] Mochizuki T., Matsukawa M., Tanaka T., Jiang H. (2024). Initial eGFR Changes Predict Response to Tolvaptan in ADPKD. Kidney360.

[29] Ozkok A., Akpinar T.S., Tufan F., Kanitez N.A., Uysal M., Guzel M., Caliskan Y., Alisir S., Yazici H., Ecder T. (2013). Clinical characteristics and predictors of progression of chronic kidney disease in autosomal dominant polycystic kidney disease: A single center experience. Clin. Exp. Nephrol..

[30] Gansevoort R.T., Meijer E., Chapman A.B., Czerwiec F.S., Devuyst O., Grantham J.J., Higashihara E., Krasa H.B., Ouyang J., Perrone R.D. (2016). Albuminuria and tolvaptan in autosomal-dominant polycystic kidney disease: Results of the TEMPO 3:4 Trial. Nephrol. Dial. Transplant..

[31] Bailey C.J., Day C., Bellary S. (2022). Renal Protection with SGLT2 Inhibitors: Effects in Acute and Chronic Kidney Disease. Curr. Diabetes Rep..

[32] Minatoguchi S., Hayashi H., Umeda R., Koide S., Hasegawa M., Tsuboi N. (2024). Additional renoprotective effect of the SGLT2 inhibitor dapagliflozin in a patient with ADPKD receiving tolvaptan treatment. CEN Case Rep..

[33] Nishida J., Yamakawa M., Miura S., Yasutomi M. (2025). Renoprotective effects of combination therapy with tolvaptan and dapagliflozin in autosomal dominant polycystic kidney disease: A four-case series. CEN Case Rep..

[34] Sekine A., Hoshino J., Fujimaru T., Suwabe T., Mizuno H., Kawada M., Hiramatsu R., Hasegawa E., Yamanouchi M., Hayami N. (2020). Genetics May Predict Effectiveness of Tolvaptan in Autosomal Dominant Polycystic Kidney Disease. Am. J. Nephrol..

